# Draft Genome Sequence of *Deinococcus phoenicis*, a Novel Strain Isolated during the Phoenix Lander Spacecraft Assembly

**DOI:** 10.1128/genomeA.00301-14

**Published:** 2014-04-24

**Authors:** Victor G. Stepanov, Parag Vaishampayan, Kasthuri Venkateswaran, George E. Fox

**Affiliations:** a Department of Biology and Biochemistry, University of Houston, Houston, Texas, USA; b Biotechnology and Planetary Protection Group, Jet Propulsion Laboratory, California Institute of Technology, Pasadena, California, USA

## Abstract

*Deinococcus phoenicis* strain 1P10ME^T^ is a radiation- and desiccation-resistant bacterium isolated from a cleanroom facility where the Phoenix Lander spacecraft was assembled. In order to facilitate investigations of the nature of the extreme resistance of *D. phoenicis* to bactericidal factors, a draft genome sequence of *D. phoenicis* was determined.

## GENOME ANNOUNCEMENT

The high flux of radiation encountered during space travel and on the surfaces of Mars and Europa poses a significant challenge for the survival of microbial life. Nevertheless, some microorganisms can survive the extreme radiation and low temperature experienced during space travel and, therefore, might be transferred to these solar system bodies and even proliferate. One such organism is *Deinococcus phoenicis*, a pale-pink pigmented, tetrad-forming coccoid bacterial strain (1P10ME^T^) that was originally isolated from the Phoenix spacecraft assembly cleanroom (1). Presently, the genus *Deinococcus* comprises 50 validly published species (http://www.bacterio.net/deinococcus.html) and represents a phylogenetically diverse group. Polytaxonomic characterization of the1P10ME^T^ isolate revealed that it was only distantly related to previously described *Deinococcus* species (1). Nevertheless, *D. phoenicis* cells exhibited levels of resistance to gamma (D_10_, >8 kGy) and ultraviolet (D_10_, >1,000 J m^–2^) radiation that are comparable to those exhibited by *D. radiodurans* (2). The possible occurrence of a *D. phoenicis* population on spacecraft surfaces potentially poses a risk to the integrity of future life detection and sample return missions. Whole-genome sequencing was undertaken to better understand this organism so that spacecraft cleaning practices might be modified to eliminate it.

In this study, we determined the draft genome sequence of *D. phoenicis* strain 1P10ME^T^. Whole-genome shotgun sequencing was performed on an Illumina HiSeq 2000 instrument with a paired-end module at the University of Arizona Genetic Core Facility (Tucson, AZ). A total of 14,784,897 pairs of 100-nucleotide-long reads were collected. The reads were trimmed to remove terminal low-quality nucleotides and assembled using Velvet 1.2.10 (3) bundled with VelvetOptimiser 2.2.5 (http://bioinformatics.net.au/software.velvetoptimiser.shtml). This resulted in 94 contigs with a G+C content of 68.96% and sizes ranging from 209 to 232,991 bp (with a mean length of 40,501 bp), containing 3,807,137 bp in total. Further scaffolding of the contigs yielded 66 scaffolds encompassing 3,809,008 bp, with the largest having 353,669 bp. Protein-coding sequences (CDSs) were predicted using Glimmer 3.02 (4) and Prodigal 2.60 (5). tRNA genes were searched using tRNAscan-SE (6), while other RNA genes were found by homology search using NCBI-BLAST-2.2.29+ (7). A total of 3,558 CDSs, 47 tRNA genes, a transfer-messenger RNA gene, and an RNase P RNA gene were identified. In addition, complete 5S rRNA and 16S rRNA and partial 23S rRNA gene sequences were obtained. The predicted CDSs were evaluated using BLAST against a collection of protein genes from seven complete deinococcal genomes available at the time of this writing (*Deinococcus deserti*, *D. geothermalis*, *D. gobiensis, D. maricopensis*, *D. peraridilitoris*, *D. proteolyticus*, and *D. radiodurans*). Of a core set of 1,032 protein genes shared by all these genomes, only one was not found in the draft *D. phoenicis* genome assembly, namely a gene encoding nitric oxide synthase-like protein (DR_2597 in *D. radiodurans*). Overall, 2,654 predicted CDSs were mapped satisfactorily to deinococcal genes.

### Nucleotide sequence accession numbers.

This whole-genome shotgun project has been deposited at DDBL/EMBL/GenBank under the accession no. JHAC00000000. The version described in this paper is version JHAC01000000. The *D. phoenicis* strain has been deposited in the USDA Agricultural Research Station (NRRL B-59546^T^) and German (DSM 27173^T^) culture collections.
